# Marcus Gunn Jaw-Winking Phenomenon and Monocular Elevation Deficiency in Association With Congenital Ptosis

**DOI:** 10.7759/cureus.33817

**Published:** 2023-01-16

**Authors:** Chrisann Saldanha, Sachin Daigavane

**Affiliations:** 1 Department of Ophthalmology, Jawaharlal Nehru Medical College, Datta Meghe Institute of Higher Education & Research, Wardha, IND

**Keywords:** double elevator palsy, frontalis sling surgery, monocular elevation deficiency, marcus gunn jaw winking, : congenital ptosis

## Abstract

Marcus Gunn jaw winking (MGJW) is an uncommon entity and is associated with congenital ptosis. It is a neurogenic congenital ptosis, which is also called the Marcus Gunn phenomenon, trigeminal-oculomotor synkinesis, or pterygoid-levator synkinesis. Congenital ptosis can be associated with MGJW syndrome, blepharophimosis syndrome, and monocular elevation deficiency (MED). MED is a condition where there is a unilateral congenital abnormality in the elevation of the eye in abduction and adduction. The MGJW phenomenon, congenital ptosis, and double-elevator palsy may be associated with and represent a congenital misdirection syndrome. Together, it can be challenging, and surgery is recommended in severe cases, depending on the degree of ptosis and jaw winking. We hereby want to bring to light one such case of a 14-year-old female with congenital ptosis, MGJW, and double-elevator palsy and want to highlight how both MGJW and double-elevator palsy are both parts of the same disease spectrum and how such cases can be treated.

## Introduction

Congenital ptosis is uncommon, causes the vertical palpebral fissure to narrow, and is defined by an abnormal drooping of the upper eyelid from its anatomical position in the primary gaze that is present from birth or develops within the first year of life [1]. Congenital ptosis alone may be autosomal recessive or dominantly inherited, but it may also be a component of a wider spectrum of birth disorders when associated with other ocular or systemic problems. It often manifests as a fluctuating loosening of the upper eyelids brought on by a loss of either bilateral or unilateral muscle or nerve function [2].

Levator palpebrae superioris (LPS) muscle dysgenesis, where there is the replacement of healthy muscle fibers with adipose and fibrous tissue, is the cause of congenital ptosis. It can also happen due to LPS muscle innervation being stopped, which would make it a type of congenital cranial dysinnervation condition [3]. It may be associated with the Marcus Gunn jaw-winking (MGJW) syndrome, where there is synkinesis between the upper eyelid and side-to-side jaw movement due to an abnormal association among the motor branches of the fifth (trigeminal) nerve and superior part of the oculomotor nerve. It was initially noted in 1883 by a Scottish ophthalmologist Dr. Robert Marcus Gunn [1].

He showed that ptosis improved with the side-to-side movement of the pterygoid muscles of the jaw. Around 2% to 13% of newborns with congenital ptosis show the MGJW phenomenon [1]. Double-elevator palsy or monocular elevation deficiency (MED) is a condition with a defect in the elevation of one eye in abduction and adduction. It could be caused by superior rectus muscle paralysis, inferior rectus muscle limitation, or a supranuclear problem [3]. Congenital ptosis, the Marcus Gunn phenomenon, and double-elevator palsy may be associated with and represent a congenital misdirection syndrome. Together, it can be challenging, and surgery is recommended in severe cases, depending on the degree of ptosis and jaw winking.

## Case presentation

A 14-year-old female came to the Ophthalmology Outpatient Department with complaints of drooping of the left upper eyelid, nonprogressive and stationary. She did not give any history of trauma, difficulty in swallowing, diplopia, or generalized weakness. She was born from a full-term normal vaginal delivery from healthy parents, with no history of staying in the neonatal intensive care unit. Neurological examination was within normal limits. All developmental milestones were achieved at the right time, and she had no past medical or surgical history. The visual acuity of both eyes was 6/6, pupils were reactive to light, and the lens did not show any abnormality. Posterior segment evaluation was within normal limits in both eyes.

**Figure 1:**
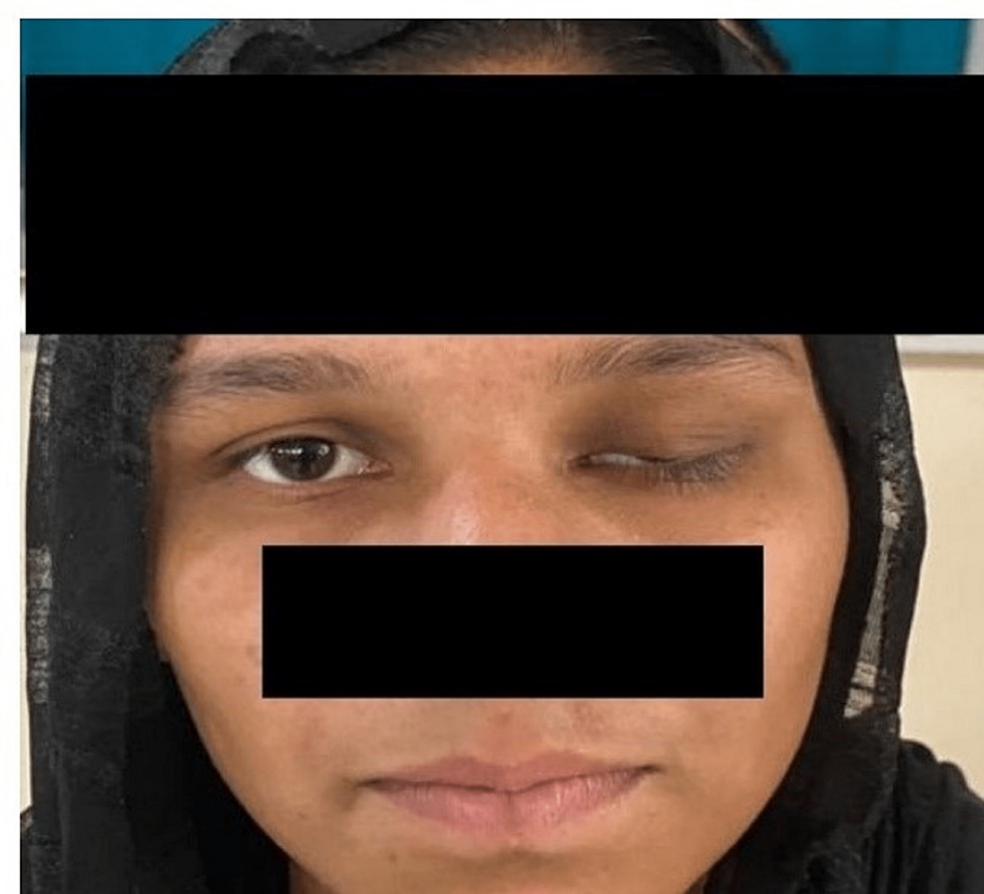
Clinical image showing severe congenital ptosis in the left eye.

**Figure 2:**
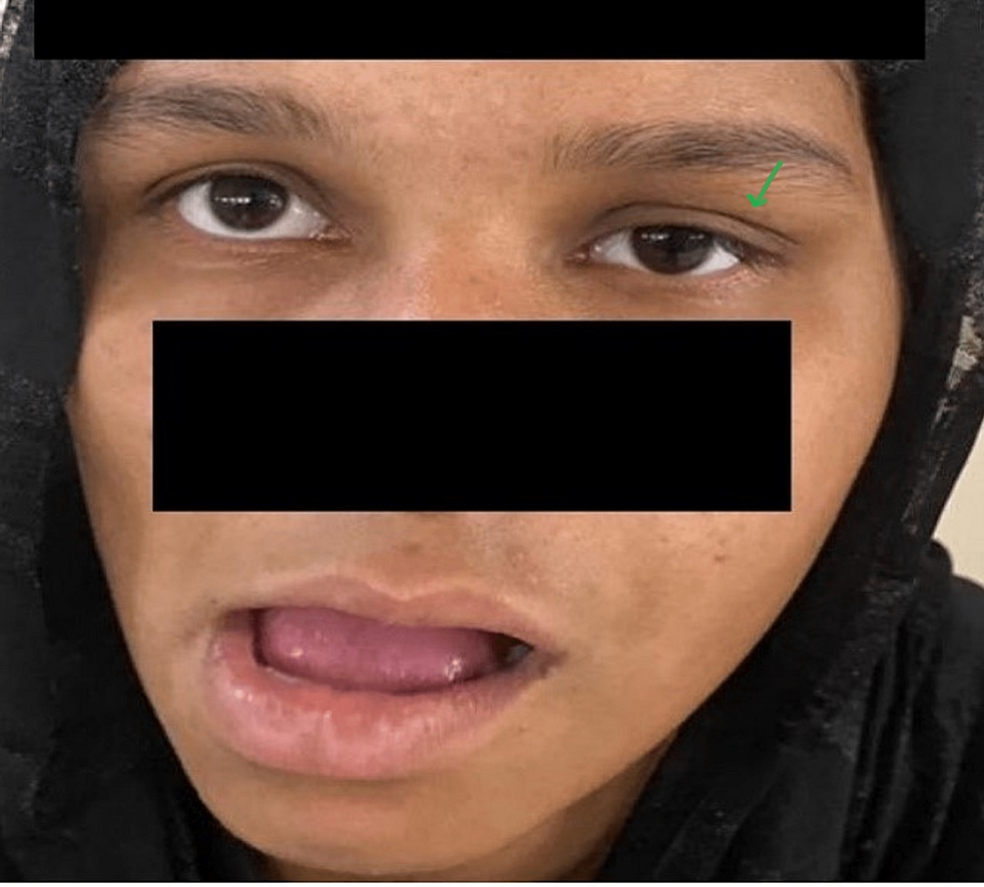
Clinical image showing elevation of the left upper eyelid (ptotic) on the side-to-side movement of the pterygoid muscles of the jaw.

The right eye showed the presence of the lid crease, which was absent in the left eye, and chin elevation was present with frontalis overaction. The left eye showed the presence of severe ptosis of 6 mm. In the right eye, the marginal reflex distance 1 was 5 mm, in the left eye, it was -3 mm. In the right and left eyes, the marginal reflex distance 2 values were 5 and 5 mm, respectively. The palpebral aperture's height was 2 mm in the left eye and 10 mm in the right. The levator palpebrae superioris (LPS) function test results for the right eye were normal (15 mm) and that for the left eye was poor (3 mm), both with good Bell's phenomenon. The patient's ptosis improved when she moved her jaw to the right side (normal side), and her eyelid opened to about 8 mm. However, when she moved her jaw to the left, her eyelid did not change (Figures 1-2). Extraocular muscle movements showed restriction in dextroelevation and levoelevation in the left eye. Ice pack test showed no improvement in ptosis.

**Figure 3:**
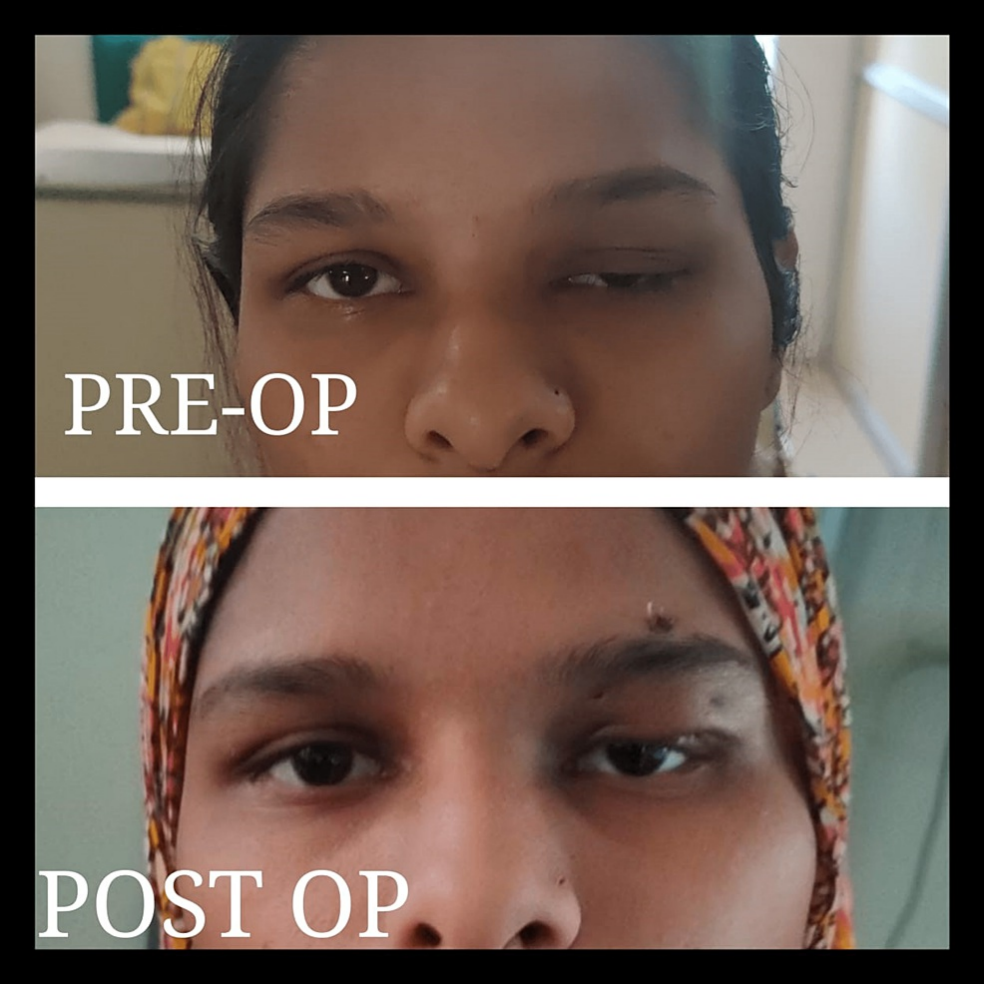
Clinical image showing the preoperative and postoperative images of the patient after the frontalis sling suspension procedure.

As the patient had poor LPS function with severe ptosis and good Bell’s phenomenon, she was managed with the help of frontalis sling surgery in the left eye (Figure 3). Systemic examination and posterior segment evaluation were both within normal limits. The procedure was performed under general anesthesia. To outline the intended skin incisions, a marking pen was used. The Fox pentagon links the upper eyelid's tarsus to the frontalis muscle, improving the position of the eyelids during primary gaze. Under general anesthesia, supralash stab incisions were made considering the skin and orbicularis muscle. Using the Wright's ptosis surgery needle, a silicon nasal intubation tube was threaded beneath the skin and orbicularis. The top of the pentagon's stab incision was made through the skin and frontalis muscle, and the two ends of the silicon tube were brought out in line with the center of the pupil. The ptotic lid was lifted to a position slightly below the superior limbus by tightly bringing the silicon tube's two ends together and tying them together in a temporary knot. At the tip of the pentagon, a little subcutaneous pocket was made to house the knot and transfixing 6/0 Prolene suture. Frost suture was applied to prevent exposure to keratopathy. The patient was then pressure-padded for 24 hours and then examined.

## Discussion

Ptosis is a Greek term that means *to droop*. The upper eyelid border is normally 1 to 2 mm below the superior limbus. Ptosis can be inherited or acquired. It might be unilateral or bilateral in nature. Congenital ptosis severely impacts the physiological development of normal vision, resulting in amblyopia and cervical spine abnormalities, particularly in bilateral cases with compensatory chin-up head posture [1,4]. Thus, congenital ptosis should be rectified in childhood, and amblyopia treatment should begin as soon as the diagnosis is established. Although the cause is uncertain, congenital ptosis is caused by levator function developmental dystrophy. It could be associated with third nerve misdirection, MGJW, or blepharophimosis syndrome [5].

Upper eyelid retraction and pterygoid muscle movement occur simultaneously in MGJW, a syndrome caused by nerve dysfunction. It is accompanied by ptosis of the upper eyelid in various degrees. Both eyes and sexes have an identical prevalence of MGJW. Many times, MGJW is unilateral [6]. It usually occurs on its own, but in a few instances, it coexists with other ocular or systemic disorders such as iris heterochromia, Duane's syndrome, upper rectus muscle paralysis, and pseudo inferior oblique overactivity. Chewing, lateral mandibular movement, smile, contraction of the sternocleidomastoid muscle, suction, tongue protrusion, Valsalva maneuver, and even breathing will elevate and even withdraw the affected eyelid in the Marcus Gunn phenomenon. The cause of this is unclear. The genesis of this illness has been the subject of various theories. According to the first explanation, there is a disruption in the levator muscle's innervation from the external pterygoid part of the trigeminal nucleus and the oculomotor nucleus [5,6].

Only the oculomotor nucleus innervates this muscle in its normal. A reflex arc traveling from the trigeminal nervous system to the Gasserian ganglion is described in the second theory of nerve. It continues past the neural connections to the oculomotor nucleus and eventually travels to reach the muscle of the upper eyelid. The nuclei of the two cranial nerves are closely spaced at a time; this could explain the above theories. In the absence of nerve supply, the third nerve's distal end favors the portion of the superior rectus muscle over the levator palpebral muscle [7]. If the nerve supply is higher, both LPS and superior rectus contract, the MGJW and MED are present simultaneously, and the upper rectus muscle is entirely supplied. Both of these muscles become active when the innervation is weaker. Levator and superior rectus muscle complexes were not innervated, which resulted in MGJW. This condition most likely falls under congenital cerebral dysinnervation diseases [7].

As we all know, a unilateral defect in abduction and adduction of the eye in elevation since birth results in double-elevator palsy or monocular elevation insufficiency. It could be caused by a supranuclear issue, inferior rectus muscular limitation, superior rectus muscle palsy, or both. Given that the patient and her aunt most likely have the same ancestor, MED was exhibited in the adolescent as severe ptosis, whereas MGJW was expressed in her aunt as milder ptosis; double-elevator palsy and MGJW seem to be two extreme examples of the same disease. Surgical options in these cases completely depend on the amount and degree of ptosis and MGJW [8]. It is a calculative decision to be taken by the surgeon, keeping the patient’s best interest in mind. However, more studies should be conducted to study the management of such cases.

## Conclusions

MGJW and double-elevator palsy can be part of the same disease spectrum with varying degrees of severity. Future studies will, however, need to look at more cases and concentrate on the relationship between MED and the severity of MGJW, as well as the level of ptosis and prevalence of MED. In treating these individuals, an early diagnosis should be made to provide appropriate, prompt, and individually tailored treatment based on the severity and co-occurring disorders.
